# The Emerging Role of lncRNAs in Spinal Cord Injury

**DOI:** 10.1155/2019/3467121

**Published:** 2019-10-15

**Authors:** Fei Wang, Junzhi Liu, Xiunan Wang, Jigang Chen, Qingjie Kong, Baoguo Ye, Zhenxing Li

**Affiliations:** ^1^Department of Orthopedics, China-Japan Union Hospital, Jilin University, Changchun, Jilin, China; ^2^Quality Control Department, China-Japan Union Hospital, Jilin University, Changchun, Jilin, China; ^3^Department of Orthopedics, The 964th Hospital of the PLA Joint Logistics Support Force, Changchun, Jilin, China; ^4^Department of Neurosurgery, Changzheng Hospital, Second Military Medical University, Shanghai, China; ^5^Department of Orthopedic Surgery, Spine Center, Changzheng Hospital, Second Military Medical University, Shanghai, China; ^6^Department of Anesthesiology, China-Japan Union Hospital, Jilin University, Changchun, Jilin, China

## Abstract

Spinal cord injury (SCI) is a highly debilitating disease and is increasingly being recognized as an important global health priority. However, the mechanisms underlying SCI have not yet been fully elucidated, and effective therapies for SCI are lacking. Long noncoding RNAs (lncRNAs), which form a major class of noncoding RNAs, have emerged as novel targets for regulating several physiological functions and mediating numerous neurological diseases. Notably, gene expression profile analyses have demonstrated aberrant changes in lncRNA expression in rats or mice after traumatic or nontraumatic SCI. LncRNAs have been shown to be associated with multiple pathophysiological processes following SCI including inflammation, neural apoptosis, and oxidative stress. They also play a crucial role in the complications associated with SCI, such as neuropathic pain. At the same time, some lncRNAs have been found to be therapeutic targets for neural stem cell transplantation and hydrogen sulfide treatment aimed at alleviating SCI. Therefore, lncRNAs could be promising biomarkers for the diagnosis, treatment, and prognosis of SCI. However, further researches are required to clarify the therapeutic effects of lncRNAs on SCI and the mechanisms underlying these effects. In this study, we reviewed the current progress of the studies on the involvement of lncRNAs in SCI, with the aim of drawing attention towards their roles in this debilitating condition.

## 1. Introduction

Spinal cord injury (SCI) is one of the most devastating conditions that has been increasingly recognized as an important global health priority [1]. Approximately half a million people suffer from SCI injured each year [2], and there are estimated to be more than 17000 new cases of SCI annually in the United States [3]. Falls and road traffic accidents are the main contributory factors for SCI [4]. However, nontraumatic conditions, such as insufficient blood flow, inflammatory injuries, spinal tumors, and osteoarthritis, could also lead to SCI [5]. SCI survivors usually face a series of problems including paralysis, sensory dysfunction, neuropathic pain, and other dysfunctions, which pose heavy emotional and economic burdens to individuals, families, and societies [6]. Although the pathophysiological mechanism of SCI is known to be associated with the primary damage caused by initial mechanical injury and secondary damage, the exact mechanism of this disease is still unclear [7]. Secondary damage frequently occurs several minutes or months after SCI and results in persistent neurological impairment. Generally, secondary damage involves vascular dysfunction, inflammation, edema, ischemia, excitotoxicity, free radical production, and restrained apoptotic cell death [8]. Treatment strategies for SCI mainly consist of decompression, stabilization, rehabilitation, pharmacological therapies, cell transplantation, and transplantation of cells with scaffolds; these strategies vary according to the stage of the disease [8, 9]. However, there is still a lack of prompt strategies for preventing the secondary injury associated with SCI and effective treatment measures for the satisfactory recovery from SCI.

With the development of microarray and high-throughput sequence technologies, many differentially expressed genes including coding RNAs and noncoding RNAs have gradually been identified to be involved in various diseases during the past several years. Noncoding RNA genes, such as ribosomal RNAs, transfer RNAs, small nuclear RNAs, microRNAs (miRNAs), and long noncoding RNAs (lncRNAs), have been shown to exist in human and rodent genomes. MiRNAs have been recognized to be differentially expressed after SCI and may be novel biomarkers for the diagnosis, treatment, and prognosis of such injuries [10]. For example, Hachisuka et al. have analyzed candidate miRNAs whose expression levels were altered in the serum relative to the severity of SCI and identified that the serum *miR-9^∗^*, *miR-219*, and *miR-384-5p* levels may be promising biomarkers for predicting the severity of SCI [11]. LncRNAs, which are a large type of noncoding RNAs, have been emerging as important regulators of multiple disease processes [12]. However, the functional significance of lncRNAs in SCI and the mechanisms underlying their involvement are lesser known compared with those of miRNAs. LncRNAs constitute the largest portion of mammalian noncoding RNAs [13]. Once regarded as transcriptional noises, increasing evidences have demonstrated that lncRNAs have multiple domains that can bind to DNA, RNA, and proteins, modify the chromatin status, regulate transcription and translation, and ultimately affect gene expression [14]. Although the functions of lncRNAs have not yet been completely decoded, studies have shown that they play different roles in cell proliferation, survival, and migration as well as in maintaining genomic stability via guidance, bait, scaffold, and signaling molecules [15, 16]. In this review, we have summarized the current knowledge about the roles of lncRNAs associated with SCI, which would help in the search for new therapeutic strategies against SCI.

## 2. LncRNAs and Neurological Diseases

LncRNAs are highly expressed in the central nervous system (CNS) [17]. Their aberrant expression has been confirmed to be related to traumatic brain injury (TBI), neurodegenerative diseases, ischemic cerebrovascular diseases, gliomas, and other neurological diseases. Firstly, lncRNAs have been demonstrated to be significantly expressed following TBI [18, 19]. The altered expression of lncRNAs may be related to their biological functions following TBI, such as inflammatory response, metabolism, and apoptosis [20]. It is worth mentioning that lncRNAs could act as competing endogenous RNAs (ceRNAs) to regulate coding RNA transcripts by sponging miRNA [21]. For example, Yu et al. have demonstrated that the transcript of the *lncRNA Gm4419* could play the role of a sponge for *miR-466l* and promote the traumatic injury-induced apoptosis of astrocytes by upregulating the expression of the inflammatory cytokine tumor necrosis factor-alpha (TNF-*α*) [22]. Secondly, genome-wide association studies and comparative transcriptome analysis have indicated that the dysregulation of, or mutations in, lncRNA gene loci is associated with Alzheimer's disease (AD) [23], Parkinson's disease (PD) [24], and other neurodegenerative diseases [25]. For example, the level of the *lncRNA BACE1* in the cytoplasm of patients with AD has been reported to be significantly higher than that in the control patients. Its upregulation can be used as a new biomarker for the diagnosis of AD [26]. Previous studies have found that the mutations of the *UCHL1* gene and oxidative inactivation of the *Uchl1* protein were associated with familial PD [27]. The lncRNA *Uchl1-AS* has been shown to increase the protein synthesis of UCHL1 at the posttranscriptional level; this depends on the combined activities of two domains, the 50 antisense region, which confers specificity for the sense target gene, and the embedded repetitive short interspersed nuclear element of B3 subclass (SINEB2) element, which makes the protein synthesis activation domain available [28]. Thirdly, Zhang et al. have found that the knockout of the lncRNA *Malat1* in a mouse model of oxygen and glucose deprivation (OGD) led to a significant increase in the expression of proapoptotic factors and proinflammatory cytokines, indicating that *Malat1* may have a protective role against ischemic stroke [29]. Furthermore, a recent study has demonstrated that the lncRNA *SNHG6* could function as a ceRNA to regulate neuronal cell apoptosis by modulating *miR-181c-5p* and Bcl‐2‐interacting mediator of cell death signaling in ischemic stroke [30]. Lastly, the significance of lncRNAs involved in glioma has begun to be revealed. AXL, which is a receptor tyrosine kinase, has been reported to be overexpressed in human glioma and associated with poor prognosis of glioma patients [31]. Yan et al. have proved that the lncRNA *LINC00526* could repress glioma progression by forming a double negative feedback loop with AXL [32]. In addition, *MALAT1* has also been considered to decrease the sensitivity of resistant glioblastoma to chemotherapy [33]. Although many studies have recently reported the potential value of lncRNAs in neurological diseases, research regarding this topic is still in its infancy, especially with regards to their roles in SCI.

## 3. LncRNAs in SCI

### 3.1. LncRNA Expression Profiles in Traumatic SCI

LncRNA microarrays, RNA sequencing (RNA-Seq), and bioinformatics analysis are commonly used to explore the expression of lncRNAs and delineate their molecular functions. To date, in particular, six studies have reported the alterations of lncRNAs in mice or rats after traumatic SCI (Table 1). In 2015, for the first time, Wang et al. performed the screening of lncRNA expression changes after spinal cord contusion in rats at one, four, and seven days after injury [34]; seven lncRNAs were found to be differentially expressed, and *RGD1559747* (named as *lncSCIR1*) showed a significant downregulation at all time points. To better understand the molecular mechanisms of SCI and look for an early therapeutic time window, Zhou et al. have investigated the lncRNA and messenger RNA (mRNA) expression in the immediate phase after SCI [35]. A total of 772 lncRNAs and 992 mRNAs were identified two hours after the injury. The toll-like-receptor (TLR) signaling pathway, p53 signaling pathway, MAPK signaling pathway, and Jak–STAT signaling pathway have been found to be mainly associated with the differentially expressed genes. Alterations of lncRNAs at the acute stages of SCI have also been studied. Ding et al. have detected the expression patterns and levels of lncRNAs and mRNAs at five continuous time points in a mouse model of spinal cord contusion [36]. In this study, few changes in the lncRNA expression levels were observed one day after the injury. However, these changes peaked one week after SCI and subsequently declined until three weeks after SCI. Gene ontology (GO) analysis showed that the differential lncRNAs were mainly associated with transport, cell adhesion, ion transport, and metabolic processes. The Kyoto Encyclopedia of Genes and Genomes (KEGG) enrichment analysis indicated that the neuroactive ligand-receptor interaction, the PI3K-Akt signaling pathway, and focal adhesions may be involved in the pathology of SCI. Furthermore, 264 lncRNAs and 949 mRNAs have been utilized to construct a dynamic lncRNA-mRNA network to elucidate the interactions between the lncRNAs and mRNAs and predict the key modulators. Shi et al. have explored the expression of lncRNAs and mRNAs in a rat model of SCI [37]; a total of 3,193 lncRNAs and 4,308 mRNAs have been found to be differentially expressed two days after SCI. They also identified ten core genes such as IL6, TOP2A, and CDK1 using a protein-protein interaction (PPI) network. Similarly, Wang et al. have profiled the expression of noncoding RNAs and mRNAs of mice using a standard Allen's weight-drop model of SCI [38]. A total of 458 lncRNAs were identified to be differentially expressed, in addition to 498 circRNAs, 458 lncRNAs, 155 miRNAs, and 1203 mRNAs. Coexpression network analyses suggested that lncRNAs and other noncoding RNAs may play crucial roles in the acute stage of SCI, based on the patterns of the regulation of ceRNAs.

Contrary to the research in the immediate and acute stages of SCI, Duran et al. have investigated the significant alterations of lncRNAs in the subchronic and chronic stages of SCI in rats [39]. The numbers of differentially expressed lncRNAs at the one-month, three-month, and six-month time points were 137, 239, and 179, respectively. The results showed that a high level of transcriptional abnormalities persistently existed in the subchronic and chronic stages, and the altered genes were related to several pathways such as immune and inflammatory responses, as well as gliosis. This study identified a new series of lncRNAs that may potentially be involved in the progression of SCI.

The above studies have shown the dynamic lncRNA expression profiles at different stages of SCI, and these profiles were associated with the secondary injury-related pathological changes. These results provided preliminary insights into the stage‐specific regulation of lncRNAs during SCI. However, further experimental verification, such as the assessment of the effects of the knockdown or overexpression of the selected lncRNAs on SCI-induced histological changes or functional recovery at the epigenetic, transcriptional, or posttranscriptional levels, is required to confirm these aspects. Additionally, the interactions between the differentially expressed lncRNAs in SCI are yet to be discovered.

### 3.2. LncRNAs in the Pathophysiological Process of SCI

SCI, which poses a major challenge in the field of neurotrauma, triggers complex pathophysiological processes [40]. Inflammation is considered the most common phenomenon occurring immediately during the secondary injury stage of SCI, which is concomitant with the activation of microglia, T cells, and astrocytes [7, 41]. A previous study has shown that Krüppel-like factor 4 (KLF4) was involved in SCI and could regulate microglial activation and subsequent neuroinflammation [42]. In a rat model of SCI, Jiang and Zhang have observed that the lncRNA *SNHG5* and KLF4 were highly expressed during SCI [43]. They also have proved that KLF4 was a direct target for *SNHG5* and was positively regulated by *SNHG5*. *SNHG5* was further demonstrated to increase the viability of astrocytes and microglia via the upregulation of KLF4 and promote the process of SCI. Zhou et al. have also detected that the significant increase in the expression of the lncRNA *MALAT1* was accompanied by the activation of the I*κ*B kinase *β*-nuclear factor-kappa B (IKK*β*/NF-*κ*B) signaling pathway and increased expression of proinflammatory cytokines [44]. IKK*β* is a key catalytic subunit of the IKK complex and plays a crucial role in the activation of nuclear factor-kappa B (NF-*κ*B), which is a major initiator of inflammation [45]. In the spinal cord, the inhibition of NF-*κ*B activity prevents immune cells from expressing certain proinflammatory cytokines, such as interleukin-6 (IL-6), tumor necrosis factor-alpha (TNF-*α*), or IL-1*β*. Downregulation of *miR-199b* has been demonstrated to promote the process of acute SCI via the IKK*β*-NF-*κ*B signaling pathway-mediated activation of microglial cells [46]. Additionally, it has been confirmed that *MALAT1* promoted the inflammatory response of microglia following SCI via the modulation of the *miR-199b*/IKK*β*/NF-*κ*B signaling pathway. In addition, in a rat model of spinal cord ischemia reperfusion (IR) injury, Jia et al. have found that the downregulation of the lncRNA *TUG1* inhibited Toll-like receptor 4 (TLR4) signaling pathway-mediated inflammatory damage via the suppression of TLR4 interactor with leucine-rich repeats (TRIL) expression [47]. The TLR4-mediated NF-*κ*B signaling pathway is involved in spinal cord IR injury. TLR4 inhibition reduces the release of inflammatory cytokines after spinal cord IR injury [48]. TRIL, a receptor accessory protein of TLR4, plays an important role in regulating the activation of TLR4 and the release of its downstream inflammatory cytokine IL-1*β* [49]. Therefore, the inhibition of TLR4 by the lncRNA *TUG1* would reduce the release of inflammatory cytokines after spinal cord IR injury [48]. Moreover, in a rat model of compressive SCI, Yu et al. have found that the overexpression of the lncRNA *TUSC7* could have inhibited microglial activation and the expression of inflammatory factors in microglial cells by regulating the expression of peroxisome proliferator-activated receptor gamma through *miR-449a* [50]. Therefore, these studies provided valuable insights into the role of lncRNAs in the neuroinflammatory processes associated with traumatic or nontraumatic SCI.

Neuronal apoptosis is also an important feature in the pathophysiology of SCI [51]. Gu et al. have evaluated the roles of the lncRNA X-inactive specific transcript (*XIST*) in the pathogenesis of SCI [52]. They have found that the downregulation of *XIST* contributed to the suppression of neuronal apoptosis and the protective effects of *XIST* knockdown relied on a sponge for *miR-494*, which ultimately regulated the phosphatase and tensin homolog deleted on chromosome ten (PTEN)/phosphoinositide 3-OH kinase (PI3K)/AKT signaling pathway. More specifically, PTEN can restrict the binding of AKT to plasma membranes, thereby decreasing its activity [53]. The PI3K/AKT signaling pathway is a major determinant of the control of diverse cellular processes, including SCI [54]. PI3K activates phosphorylation and results in the activation of AKT. AKT has important downstream effects such as localizing FOXO in the cytoplasm, activating CREB [55], and activating mTOR, subsequently affecting the transcription of either p70 or 4EBP1 [56]. This pathway is crucial in facilitating both the growth and proliferation of adult neural cells. Analogously, the knockdown of the lncRNA brain-derived neurotrophic factor antisense (*lncRNA BDNF-AS*) has been identified to suppress neuronal cell apoptosis by sponging *miR-130b-5p* and targeting gene (*PRDI-BF1* and *RIZ*) domain protein 5 [57]. Different from the protective effects caused by the knockdown of some specific lncRNAs, the overexpression of the lncRNA *Map2k4* and *TCTN2* has been found to protect neurons from apoptosis after SCI by regulating the *miR-199a*/FGF1 pathway and enhancing cell autophagy, respectively [58, 59]. FGF1 is a multifunctional peptide growth factor and is mainly distributed in the CNS [60]. The FGF1 pathway is essential for the survival of neurons and plays an important role in CNS injury repair. Although further researches are needed to verify the therapeutic value of lncRNAs, these studies indicate some potential treatment targets for SCI.

**Figure 1:**
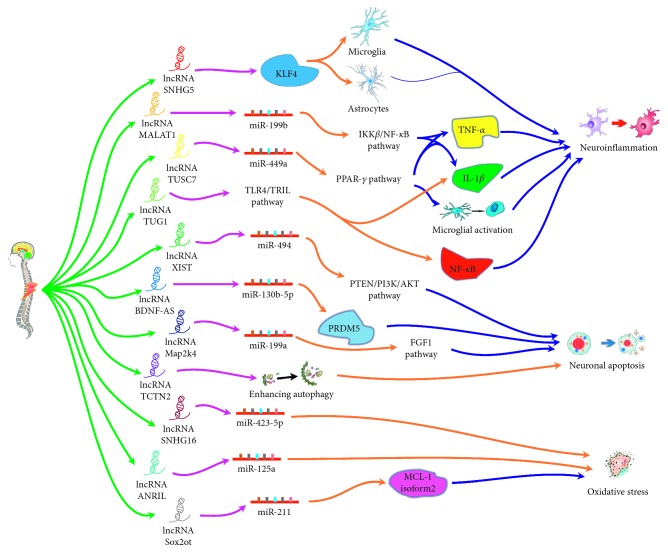
The probable mechanisms of the effects produced by lncRNAs on SCI. There are several lncRNAs that have been reported to play different roles mainly in the process of neuroinflammation, neuronal apoptosis, and oxidative stress following SCI. Firstly, lncRNA *SNHG5* was demonstrated to increase the viability of microglia and astrocytes via upregulating KLF4. lncRNA *MALAT1* overexpression could cause the activation of IKK*β*/NF-*κ*B signaling pathway via the modulation of a *miR-199b* and increased proinflammatory cytokines. Overexpression of lncRNA *TUSC7* could inhibit microglial activation and the expression of inflammatory factors in microglia cells by regulating PPAR-*γ* through *miR-449a*. Downregulation of lncRNA *TUG1* inhibited TLR4 signaling pathway-mediated inflammatory damage via suppressing TRIL expression. Secondly, downregulation of lncRNA *XIST* contributed to the limiting neuronal apoptosis via as a sponge for *miR-494* which ultimately regulated PTEN/PI3K/AKT signaling pathway. Knockdown of lncRNA *BDNF-AS* was identified to suppress neuronal cell apoptosis by sponging *miR-130b-5p* and targeting gene PRDM5. Overexpression of lncRNA *Map2k4* and lncRNA *TCTN2* could reduce apoptosis after SCI by regulating a *miR-199a*/FGF1 pathway and enhancing cell autophagy, respectively. Thirdly, overexpressed lncRNA *SNHG16* could alleviate H_2_O_2_-induced cell injury by mediating *miR-423-5p* in PC-12 cells. Suppression of lncRNA *ANRIL* might aggravate H_2_O_2_-induced injury in PC-12 cells by targeting *miR-125a*. However, downregulation of lncRNA *Sox2ot* might relieve H_2_O_2_ injury in PC-12 cells via regulating the *miR-21*-myeloid cell leukemia-1 isoform2 axis.

Oxidative stress generally proceeds after the occurrence of SCI; it would contribute to tissue injury and cell apoptosis during the secondary injury stage of SCI [61, 62]. In some researches [63–66], pheochromocytoma (PC-12) cells cultured under a condition of hydrogen peroxide (H_2_O_2_) stimulation have been used as a common in vitro cell-damage model to simulate secondary oxidative stress changes after SCI. In the study by Liu et al., the overexpression of the lncRNA small nucleolar RNA host gene 16 (*SNHG16*) has been shown to alleviate H_2_O_2_-induced cell injury by regulating the expression of *miR-423-5p* in PC-12 cells [64]. Similar to the protective effects of *SNHG16*, suppression of the lncRNA *ANRIL* may aggravate H_2_O_2_-induced injury in PC-12 cells by targeting *miR-125*a [65]. However, the lncRNA Sox2 overlapping transcript (*Sox2ot*) might enhance oxidative stress, given the observation that the downregulation of *Sox2ot* has been shown to alleviate H_2_O_2_ injury in PC-12 cells by regulating the *miR-211*-myeloid cell leukemia-1 isoform2 axis [66]. The different roles of lncRNAs in the pathophysiological process of SCI are summarized in Figure 1.

### 3.3. LncRNAs in the Complications of SCI

Neuropathic pain (NP) can develop after nerve injuries and is mostly defined as pain associated with a lesion or disease of the somatosensory nervous system [67, 68]. Approximately 30–50% of SCI patients have NP, which is one of the most common complications of SCI [69]. Refractory diseases such as NP are a worldwide health problem. Functional roles of lncRNAs and their potential mechanisms in NP have been reviewed in the previous literatures [70, 71]. The levels of lncRNAs have been reported to be significantly altered in the spinal cord of an animal model of spared nerve injury [72, 73]. The expression profiling of lncRNAs in dorsal root ganglia has also revealed that the expression of lncRNAs is dysregulated after nerve injury [74]. GO analysis has shown that several lncRNAs were enriched for sensory and neuronal processes [75]. Additionally, Hu et al. have reported that the silencing of the lncRNA *PKIA-AS1* could attenuate NP via the downregulation of CDK6 expression in the spinal cord tissues of a rat model of spinal nerve ligation [76]. In a rat model of chronic constriction injury, the lncRNAs *NEAT1* and *XIST* have also been identified to contribute to the development of NP by targeting the *miR-381*/HMGB1 axis or regulating *miR-150* and ZEB1, respectively [77, 78]. In summary, extensive genomic evidence has been provided for the novel potential targets of lncRNAs for the treatment of NP.

### 3.4. LncRNAs in the Treatment of SCI

In recent years, stem cells have gained plenty of attentions due to their immense potential of self-renewal and generating cells from glial and neuronal lineages [79]. Neural stem cells have been proposed as a promising therapeutic method for treating SCI [80]. However, the clinical transformation and application of stem cells for the treatment of SCI still have a long way to go. Fortunately, recent studies have indicated that lncRNAs play vital roles in the self-renewal and determination of the fate of stem cells [81, 82]. In particular, the lncRNA human urothelial carcinoma associated 1 (*UCA1*) has been demonstrated to regulate the proliferation and differentiation of neural stem cells by controlling the *miR-1*/Hes1 expression [83]. This study implicated important roles of lncRNAs in neural stem cells for the repair of SCI.

Hydrogen sulfide has been recognized as an important neuromodulator and neuroprotective agent for SCI [84, 85]. However, the mechanisms underlying the protective effects of hydrogen sulfide against SCI are still unclear. Liu et al. have found that the expression of the lncRNA cancer susceptibility candidate 7 (*CasC7*) was significantly reduced in rats with spinal cord I/R injury and in vitro [86]. However, hydrogen sulfide preprocessing has been shown to upregulate *CasC7* and downregulate miR-30c in vitro. Silencing of *CasC7* could upregulate *miR-30c* expression and promote cell apoptosis. Further, this study also demonstrated that hydrogen sulfide could upregulate *CasC7* expression and subsequently and protect rats from spinal cord I/R injury. This study presents a novel direction in the search for drug targets for the treatment of SCI.

### 3.5. MiRNAs and SCI

Since many lncRNAs exert their functions via regulating miRNAs, the relationship between miRNAs and SCI should be investigated to identify more targeted therapies for SCI. MiRNAs are endogenous small noncoding RNAs composed of ∼22 nt nucleotides that can function as gene regulators by binding to the 3′-untranslated region (UTR) of target miRNAs [87]. A great number of miRNAs have been found in the spinal cord, and some miRNAs are specialized for specific cell types [88, 89]. Microarray studies have indicated that the expression of miRNAs was significantly altered after SCI, and these alterations had important effects on the pathogenesis of secondary SCI [88, 90]. Bcl-2 family proteins, including proapoptotic BH3-only family members and myeloid cell leukemia sequence-1 (Mcl-1), can mediate neuronal apoptosis. *MiR-20a* and *miR-29b* have been confirmed to target the Mcl-1 and BH3-only family genes, respectively, and together, they regulate SCI-induced apoptosis [91, 92]. *MiR-21* expression has been shown to be upregulated following SCI, and *miR-21* has been demonstrated to participate in the regulation of astrocytic and glial hypertrophy after SCI [93]. The *miR-223* expression has been shown to have increased after SCI; it may regulate the activity of neutrophils and macrophages in the secondary damage phase of SCI [94]. Furthermore, miRNAs may also be used as biomarkers for diagnostic and prognostic purposes with regards to SCI [95]. Due to multiple evidences of the roles of miRNAs in SCI, a systematic analysis of the existing important miRNAs and the prediction of potential targeted lncRNAs would help elucidate the regulatory mechanisms involved following SCI.

### 3.6. Future Perspectives and Conclusion

LncRNAs have received an increasing amount of attention for their ability to modulate transcriptional regulation in neurological diseases. Several studies have reported the roles of lncRNAs after SCI. However, research regarding this aspect is currently in its infancy, and the mechanisms underlying their role in SCI are yet to be elucidated. Firstly, although alterations in lncRNA expression have been reported in SCI, more researches focused on the multidimensional interactions of lncRNAs that are not just limited to their functions as ceRNAs, and more comprehensive analyses of their expression profiles, such as analyses involving their coupling with circular RNAs, are needed. Secondly, alterations in the expression levels of lncRNAs in blood samples after SCI have not yet been studied. Such lncRNAs with abnormal findings in blood tests may serve as potential novel biomarkers. Thirdly, although the roles of several lncRNAs in the pathophysiological process and treatment of SCI have been explored, more profound researches are relatively scarce, and the validation of their functions is not powerful enough.

To better understand the roles of lncRNAs in SCI, we have several suggestions for potential further studies. As a series of lncRNAs have been identified following SCI, their respective functions could be determined by investigating which molecules (DNA, RNA, or proteins) and these lncRNAs may potentially interact with. Some technologies have been developed to validate the molecular mechanisms of lncRNAs. For example, RNA antisense purification (RAP), chromatin isolation by RNA purification-sequencing (ChIRP), and capture hybridization analysis of RNA targets (CHART) are popular methods to identify target genes of lncRNAs. RNA pull-down assays, RNA immunoprecipitation followed by high-throughput sequencing (RIP-seq), and RAP-mass spectrometry (RAP-MS) are also commonly used to explore the interactions between lncRNAs and proteins [96]. In addition, the recent single-cell RNA-Seq technique provides a new opportunity for explaining the roles of lncRNAs at the single-cell resolution to understand their cell-specific mechanisms.

In conclusion, lncRNAs have been recognized to play multiple complex roles in SCI and may subsequently serve as promising biomarkers for the diagnosis, evaluation, treatment, and prognosis of this debilitating disease. Future studies focused on the detailed mechanisms, and functional roles of lncRNAs are required to develop targeted therapies for SCI.

## Figures and Tables

**Table 1:** The differential expression profile of lncRNAs after SCI.

Published year	Animal	Model	Injury sites	Time point	Methods	Differentially expressed lncRNAs	Reference
2015	Rats	Contusion SCI	T10	1, 4, and 7 d	RNA sequencing	7 (lncSCIR1 constantly downregulated)	Wang et al. [34]
2016	Mice	Contusion SCI	T10	1 d, 3 d, 1 w, and 3 w	Microarray	Few changes in 1 d, peaked in 1 w and declined in 3 w	Ding et al. [36]
2016	Rats	SCI	T9	1 m, 3 m and 6 m	RNA sequencing	1 m: 137 (120 up, 17 down); 3 m: 239 (162 up, 77 down); 6 m: 179 (125 up, 54 down)	Duran et al. [39].
2017	Rats	Contusion SCI	T10	2 h	Microarray	772 (528 up, 244 down)	Zhou et al. [35]
2018	Rats	Contusion SCI	T10	2 d	Microarray	3193 (1332 up, 1,861 down)	Shi et al. [37]
2019	Mice	Standard Allen's weight-drop model of SCI	T8-10	3 d	RNA sequencing	458 (356 up, 93 down)	Wang et al. [38]

d, day; h, hour; w, week; lncRNAs, long noncoding RNAs; m: month; SCI, spinal cord injury; T: thoracic level.

## References

[1] Collaborators GTBIaSCI (2019). Global, regional, and national burden of traumatic brain injury and spinal cord injury, 1990–2016: a systematic analysis for the Global Burden of Disease Study 2016. *Lancet Neurology*.

[2] Holmes D. (2017). Spinal-cord injury: spurring regrowth. *Nature*.

[3] Tran A. P., Warren P. M., Silver J. (2018). The biology of regeneration failure and success after spinal cord injury. *Physiological Reviews*.

[4] Badhiwala J. H., Wilson J. R., Fehlings M. G. (2019). Global burden of traumatic brain and spinal cord injury. *The Lancet Neurology*.

[5] Hatch B. B., Wood-Wentz C. M., Therneau T. M., Walker M. G., Payne J. M., Reeves R. K. (2017). Factors predictive of survival and estimated years of life lost in the decade following nontraumatic and traumatic spinal cord injury. *Spinal Cord*.

[6] Ramer L. M., Ramer M. S., Bradbury E. J. (2014). Restoring function after spinal cord injury: towards clinical translation of experimental strategies. *The Lancet Neurology*.

[7] Venkatesh K., Ghosh S. K., Mullick M., Manivasagam G., Sen D. (2019). Spinal cord injury: pathophysiology, treatment strategies, associated challenges, and future implications. *Cell and Tissue Research*.

[8] Rowland J. W., Hawryluk G. W. J., Kwon B., Fehlings M. G. (2008). Current status of acute spinal cord injury pathophysiology and emerging therapies: promise on the horizon. *Neurosurgical Focus*.

[9] Koffler J., Zhu W., Qu X. (2019). Biomimetic 3D-printed scaffolds for spinal cord injury repair. *Nature Medicine*.

[10] Li F., Zhou M.-W. (2019). MicroRNAs in contusion spinal cord injury: pathophysiology and clinical utility. *Acta Neurologica Belgica*.

[11] Hachisuka S., Kamei N., Ujigo S., Miyaki S., Yasunaga Y., Ochi M. (2014). Circulating microRNAs as biomarkers for evaluating the severity of acute spinal cord injury. *Spinal Cord*.

[12] Iyer M. K., Niknafs Y. S., Malik R. (2015). The landscape of long noncoding RNAs in the human transcriptome. *Nature Genetics*.

[13] Mercer T. R., Dinger M. E., Mattick J. S. (2009). Long non-coding RNAs: insights into functions. *Nature Reviews Genetics*.

[14] Batista P. J., Chang H. Y. (2013). Long noncoding RNAs: cellular address codes in development and disease. *Cell*.

[15] Rinn J. L., Chang H. Y. (2012). Genome regulation by long noncoding RNAs. *Annual Review of Biochemistry*.

[16] Kung J. T. Y., Colognori D., Lee J. T. (2013). Long noncoding RNAs: past, present, and future. *Genetics*.

[17] Ng S.-Y., Lin L., Soh B. S., Stanton L. W. (2013). Long noncoding RNAs in development and disease of the central nervous system. *Trends in Genetics*.

[18] Wang C.-f., Zhao C.-c., Weng W.-j. (2017). Alteration in long non-coding RNA expression after traumatic brain injury in rats. *Journal of Neurotrauma*.

[19] Li Z., Han K., Zhang D., Chen J., Xu Z., Hou L. (2019). The role of long noncoding RNA in traumatic brain injury. *Neuropsychiatric Disease and Treatment*.

[20] Zhong J., Jiang L., Cheng C. (2016). Altered expression of long non-coding RNA and mRNA in mouse cortex after traumatic brain injury. *Brain Research*.

[21] Tay Y., Rinn J., Pandolfi P. P. (2014). The multilayered complexity of ceRNA crosstalk and competition. *Nature*.

[22] Yu Y., Cao F., Ran Q., Wang F. (2017). Long non-coding RNA Gm4419 promotes trauma-induced astrocyte apoptosis by targeting tumor necrosis factor *α*. *Biochemical and Biophysical Research Communications*.

[23] Lee D. Y., Moon J., Lee S.-T. (2015). Distinct expression of long non-coding RNAs in an Alzheimer’s disease model. *Journal of Alzheimer’s Disease*.

[24] Kraus T. F. J., Haider M., Spanner J., Steinmaurer M., Dietinger V., Kretzschmar H. A. (2017). Altered long noncoding RNA expression precedes the course of Parkinson’s disease-a preliminary report. *Molecular Neurobiology*.

[25] Quan Z., Zheng D., Qing H. (2017). Regulatory roles of long non-coding RNAs in the central nervous system and associated neurodegenerative diseases. *Frontiers in Cellular Neuroscience*.

[26] Feng L., Liao Yu-T., He J.-C. (2018). Plasma long non-coding RNA BACE1 as a novel biomarker for diagnosis of Alzheimer disease. *BMC Neurology*.

[27] Choi J., Levey A. I., Weintraub S. T. (2004). Oxidative modifications and down-regulation of ubiquitin carboxyl-terminal hydrolase L1 associated with idiopathic Parkinson’s and Alzheimer’s diseases. *Journal of Biological Chemistry*.

[28] Carrieri C., Cimatti L., Biagioli M. (2012). Long non-coding antisense RNA controls Uchl1 translation through an embedded SINEB2 repeat. *Nature*.

[29] Zhang X., Tang X., Liu K., Hamblin M. H., Yin K.-J. (2017). Long noncoding RNA Malat1 regulates cerebrovascular pathologies in ischemic stroke. *The Journal of Neuroscience*.

[30] Zhang X. a., Liu Z., Shu Q., Yuan S., Xing Z., Song J. (2019). LncRNA SNHG6 functions as a ceRNA to regulate neuronal cell apoptosis by modulating miR-181c-5p/BIM signalling in ischaemic stroke. *Journal of Cellular and Molecular Medicine*.

[31] Hutterer M., Knyazev P., Abate A. (2008). Axl and growth Arrest specific gene 6 are frequently overexpressed in human gliomas and predict poor prognosis in patients with glioblastoma multiforme. *Clinical Cancer Research*.

[32] Yan J., Xu C., Li Y. (2019). Long non-coding RNA LINC00526 represses glioma progression via forming a double negative feedback loop with AXL. *Journal of Cellular and Molecular Medicine*.

[33] Li H., Yuan X., Yan D. (2017). Long non-coding RNA MALAT1 decreases the sensitivity of resistant glioblastoma cell lines to temozolomide. *Cellular Physiology and Biochemistry*.

[34] Wang J., Hu B., Cao F., Sun S., Zhang Y., Zhu Q. (2015). Down regulation of lncSCIR1 after spinal cord contusion injury in rat. *Brain Research*.

[35] Zhou H., Shi Z., Kang Y. (2018). Investigation of candidate long noncoding RNAs and messenger RNAs in the immediate phase of spinal cord injury based on gene expression profiles. *Gene*.

[36] Ding Y., Song Z., Liu J. (2016). Aberrant LncRNA expression profile in a contusion spinal cord injury mouse model. *BioMed Research International*.

[37] Shi Z., Ning G., Zhang B. (2019). Signatures of altered long noncoding RNAs and messenger RNAs expression in the early acute phase of spinal cord injury. *Journal of Cellular Physiology*.

[38] Wang W., Su Y., Tang S. (2019). Identification of noncoding RNA expression profiles and regulatory interaction networks following traumatic spinal cord injury by sequence analysis. *Aging*.

[39] Duran R. C., Yan H., Zheng Y. (2017). The systematic analysis of coding and long non-coding RNAs in the sub-chronic and chronic stages of spinal cord injury. *Scientific Reports*.

[40] Albayar A. A., Roche A., Swiatkowski P. (2019). Biomarkers in spinal cord injury: prognostic insights and future potentials. *Frontiers in Neurology*.

[41] Ahuja C. S., Nori S., Tetreault L. (2017). Traumatic spinal cord injury-repair and regeneration. *Neurosurgery*.

[42] Choi D. K., Gupta M., Das S., Basu A. (2010). Krüppel-like factor 4, a novel transcription factor regulates microglial activation and subsequent neuroinflammation. *Journal of Neuroinflammation*.

[43] Jiang Z.-S., Zhang J.-R. (2018). LncRNA SNHG5 enhances astrocytes and microglia viability via upregulating KLF4 in spinal cord injury. *International Journal of Biological Macromolecules*.

[44] Zhou H.-J., Wang L.-Q., Wang D.-B. (2018). Long noncoding RNA MALAT1 contributes to inflammatory response of microglia following spinal cord injury via the modulation of a miR-199b/IKK*β*/NF-*κ*B signaling pathway. *American Journal of Physiology-Cell Physiology*.

[45] Perkins N. D. (2007). Integrating cell-signalling pathways with NF-*κ*B and IKK function. *Nature Reviews Molecular Cell Biology*.

[46] Zhou H.-J., Wang L.-Q., Xu Q.-S. (2016). Downregulation of miR-199b promotes the acute spinal cord injury through IKK*β*-NF-*κ*B signaling pathway activating microglial cells. *Experimental Cell Research*.

[47] Jia H., Ma H., Li Z. (2019). Downregulation of LncRNA TUG1 inhibited TLR4 signaling pathway-mediated inflammatory damage after spinal cord ischemia reperfusion in rats via suppressing TRIL expression. *Journal of Neuropathology & Experimental Neurology*.

[48] Chang X.-Q., Wang J., Fang B., Tan W.-F., Ma H. (2014). Intrathecal antagonism of microglial TLR4 reduces inflammatory damage to blood-spinal cord barrier following ischemia/reperfusion injury in rats. *Molecular Brain*.

[49] Wochal P., Rathinam V. A. K., Dunne A. (2014). TRIL is involved in cytokine production in the brain following *Escherichia coli* infection. *The Journal of Immunology*.

[50] Yu Y., Zhu M., Zhao Y., Xu M., Qiu M. (2018). Overexpression of TUSC7 inhibits the inflammation caused by microglia activation via regulating miR-449a/PPAR-*γ*. *Biochemical and Biophysical Research Communications*.

[51] Zhao W., Li H., Hou Y., Jin Y., Zhang L. (2019). Combined Administration of poly-ADP-ribose polymerase-1 and caspase-3 inhibitors alleviates neuronal apoptosis after spinal cord injury in rats. *World Neurosurgery*.

[52] Gu S., Xie R., Liu X., Shou J., Gu W., Che X. (2017). Long coding RNA XIST contributes to neuronal apoptosis through the downregulation of AKT phosphorylation and is negatively regulated by mir-494 in rat spinal cord injury. *International Journal of Molecular Sciences*.

[53] Stambolic V., Suzuki A., de la Pompa J. L. (1998). Negative regulation of PKB/Akt-dependent cell survival by the tumor suppressor PTEN. *Cell*.

[54] Luan Y., Chen M., Zhou L. (2017). MiR-17 targets PTEN and facilitates glial scar formation after spinal cord injuries via the PI3K/Akt/mTOR pathway. *Brain Research Bulletin*.

[55] Peltier J., O’Neill A., Schaffer D. V. (2007). PI3K/Akt and CREB regulate adult neural hippocampal progenitor proliferation and differentiation. *Developmental Neurobiology*.

[56] Rafalski V. A., Brunet A. (2011). Energy metabolism in adult neural stem cell fate. *Progress in Neurobiology*.

[57] Zhang H., Li D., Zhang Y. (2018). Knockdown of lncRNA BDNF-AS suppresses neuronal cell apoptosis via downregulating miR-130b-5p target gene PRDM5 in acute spinal cord injury. *RNA Biology*.

[58] Lv H.-r. (2017). lncRNA-Map2k4 sequesters miR-199a to promote FGF1 expression and spinal cord neuron growth. *Biochemical and Biophysical Research Communications*.

[59] Ren X. D., Wan C. X., Niu Y. L. (2019). Over-expression of lncRNA TCTN2 protects neurons from apoptosis by enhancing cell autophagy in spinal cord injury. *FEBS Open Bio*.

[60] Delmas E., Jah N., Pirou C. (2016). FGF1 C-terminal domain and phosphorylation regulate intracrine FGF1 signaling for its neurotrophic and anti-apoptotic activities. *Cell Death & Disease*.

[61] Guo Z., Yuan Y., Guo Y., Wang H., Song C., Huang M. (2018). Nischarin attenuates apoptosis induced by oxidative stress in PC12 cells. *Experimental and Therapeutic Medicine*.

[62] Wang X.-J., Peng C.-H., Zhang S. (2019). Polysialic-acid-based micelles promote neural regeneration in spinal cord injury therapy. *Nano Letters*.

[63] Zompa E. A., Pizzo D. P., Hulsebosch C. E. (1993). Migration and differentiation of PC12 cells transplanted into the rat spinal cord. *International Journal of Developmental Neuroscience*.

[64] Liu H., Chen B., Zhu Q. (2019). Long non-coding RNA SNHG16 reduces hydrogen peroxide-induced cell injury in PC-12 cells by up-regulating microRNA-423-5p. *Artificial Cells, Nanomedicine, and Biotechnology*.

[65] Li R., Yin F., Guo Y.-Y., Zhao K.-C., Ruan Q., Qi Y.-M. (2017). Knockdown of ANRIL aggravates H_2_O_2_-induced injury in PC-12 cells by targeting microRNA-125a. *Biomedicine & Pharmacotherapy*.

[66] Yin D., Zheng X., Zhuang J., Wang L., Liu B., Chang Y. (2018). Downregulation of long noncoding RNA Sox2ot protects PC-12 cells from hydrogen peroxide-induced injury in spinal cord injury via regulating the miR-211-myeloid cell leukemia-1 isoform2 axis. *Journal of Cellular Biochemistry*.

[67] Attal N., Bouhassira D., Baron R. (2018). Diagnosis and assessment of neuropathic pain through questionnaires. *The Lancet Neurology*.

[68] Murnion B. P. (2018). Neuropathic pain: current definition and review of drug treatment. *Australian Prescriber*.

[69] Hatch M. N., Cushing T. R., Carlson G. D., Chang E. Y. (2018). Neuropathic pain and SCI: identification and treatment strategies in the 21st century. *Journal of the Neurological Sciences*.

[70] Wu S., Bono J., Tao Y.-X. (2019). Long noncoding RNA (lncRNA): a target in neuropathic pain. *Expert Opinion on Therapeutic Targets*.

[71] Tang S., Zhou J., Jing H. (2019). Functional roles of lncRNAs and its potential mechanisms in neuropathic pain. *Clin Epigenetics*.

[72] Zhou J., Fan Y., Chen H. (2017). Analyses of long non-coding RNA and mRNA profiles in the spinal cord of rats using RNA sequencing during the progression of neuropathic pain in an SNI model. *RNA Biology*.

[73] Liu Z., Liang Y., Wang H. (2017). LncRNA expression in the spinal cord modulated by minocycline in a mouse model of spared nerve injury. *Journal of Pain Research*.

[74] Baskozos G., Dawes J. M., Austin J. S. (2019). Comprehensive analysis of long noncoding RNA expression in dorsal root ganglion reveals cell-type specificity and dysregulation after nerve injury. *Pain*.

[75] Raju H. B., Englander Z., Capobianco E., Tsinoremas N. F., Lerch J. K. (2014). Identification of potential therapeutic targets in a model of neuropathic pain. *Frontiers in Genetics*.

[76] Hu J. Z., Rong Z. J., Li M. (2019). Silencing of lncRNA PKIA-AS1 attenuates spinal nerve ligation-induced neuropathic pain through epigenetic downregulation of CDK6 expression. *Frontiers in Cellular Neuroscience*.

[77] Xia L.-X., Ke C., Lu J.-M. (2018). NEAT1 contributes to neuropathic pain development through targeting miR-381/HMGB1 axis in CCI rat models. *Journal of Cellular Physiology*.

[78] Yan X.-T., Lu J.-M., Wang Y. (2018). XIST accelerates neuropathic pain progression through regulation of miR-150 and ZEB1 in CCI rat models. *Journal of Cellular Physiology*.

[79] Chhabra H. S., Sarda K. (2017). Clinical translation of stem cell based interventions for spinal cord injury - are we there yet?. *Advanced Drug Delivery Reviews*.

[80] Harris L., Zalucki O., Piper M., Heng J. I. (2016). Insights into the biology and therapeutic applications of neural stem cells. *Stem Cells International*.

[81] Zhou Y., Dai Q.-S., Zhu S.-C. (2016). AK048794 maintains the mouse embryonic stem cell pluripotency by functioning as an miRNA sponge for miR-592. *Biochemical Journal*.

[82] Wang L., Wu F., Song Y. (2016). Long noncoding RNA related to periodontitis interacts with miR-182 to upregulate osteogenic differentiation in periodontal mesenchymal stem cells of periodontitis patients. *Cell Death & Disease*.

[83] Jin J., Yi D., Liu Y., Wang M., Zhu Y., Shi H. (2017). Long nonding RNA UCA1 regulates neural stem cell differentiation by controlling miR-1/Hes1 expression. *American Journal of Translational Research*.

[84] Li L., Jiang H. K., Li Y. P., Guo Y. P. (2015). Hydrogen sulfide protects spinal cord and induces autophagy via miR-30c in a rat model of spinal cord ischemia-reperfusion injury. *Journal of Biomedical Science*.

[85] Liu H., Anders F., Thanos S. (2017). Hydrogen sulfide protects retinal ganglion cells against glaucomatous injury in vitro and in vivo. *Investigative Opthalmology & Visual Science*.

[86] Pfeiffer Y., Pan L., Jiang A., Yin M. (2018). Hydrogen sulfide upregulated lncRNA CasC7 to reduce neuronal cell apoptosis in spinal cord ischemia-reperfusion injury rat. *Biomedicine & Pharmacotherapy*.

[87] Bartel D. P. (2004). MicroRNAs. *Cell*.

[88] Liu N.-K., Wang X.-F., Lu Q.-B., Xu X.-M. (2009). Altered microRNA expression following traumatic spinal cord injury. *Experimental Neurology*.

[89] Bak M., Silahtaroglu A., Moller M. (2008). MicroRNA expression in the adult mouse central nervous system. *RNA*.

[90] Yunta M. ., Nieto-Díaz M., Esteban F. J. (2012). MicroRNA dysregulation in the spinal cord following traumatic injury. *PLoS One*.

[91] Liu X. J., Zheng X. P., Zhang R., Guo Y. L., Wang J. H. (2015). Combinatorial effects of miR-20a and miR-29b on neuronal apoptosis induced by spinal cord injury. *International Journal of Clinical and Experimental Pathology*.

[92] Shi Z., Zhou H., Lu L. (2017). The roles of microRNAs in spinal cord injury. *International Journal of Neuroscience*.

[93] Bhalala O. G., Pan L., Sahni V. (2012). microRNA-21 regulates astrocytic response following spinal cord injury. *Journal of Neuroscience*.

[94] Izumi B., Nakasa T., Tanaka N. (2011). MicroRNA-223 expression in neutrophils in the early phase of secondary damage after spinal cord injury. *Neuroscience Letters*.

[95] Sun P., Liu D. Z., Jickling G. C., Sharp F. R., Yin K.-J. (2018). MicroRNA-based therapeutics in central nervous system injuries. *Journal of Cerebral Blood Flow & Metabolism*.

[96] Cuevas-Diaz Duran R., Wei H., Kim D. H., Wu J. Q. (2019). Review: long non-coding RNAs: important regulators in the development, function and disorders of the central nervous system. *Neuropathology and Applied Neurobiology*.

